# Comparison of Palliative Knowledge and Self-Efficacy Expectation of German Paramedics Between a Rural and an Urban Structured Emergency Medical Service Area

**DOI:** 10.1177/08258597231221916

**Published:** 2024-02-19

**Authors:** Daniel Chwallek, Adam Schweda, Martin Neukirchen, Joachim Risse, Jörg Hense, Martin Teufel, Mitra Tewes

**Affiliations:** 1 Department of Palliative Medicine, University Hospital Essen, University of Duisburg-Essen, Essen, Germany; 2 Department of Psychosomatic Medicine and Psychotherapy, University of Duisburg-Essen, Essen, Germany; 3 Interdisciplinary Centre of Palliative Medicine, 39064 University Hospital Düsseldorf, Heinrich-Heine-University, Düsseldorf, Germany; 4 Department of Anesthesiology, 39064 University Hospital Düsseldorf, Heinrich-Heine-University, Düsseldorf, Germany; 5 Center of Emergency Medicine, 39081 University Hospital Essen, Essen, Germany; 6 West German Cancer Centre, Department of Medical Oncology, University Hospital Essen, University of Duisburg-Essen, Essen, Germany

**Keywords:** Palliative care, knowledge, paramedic, emergency medical service, end-of-Life

## Abstract

**Objective(s):** Differences in the German emergency medical service (EMS) can be seen in the countryside in contrast to the city with regard to travel distances to hospitals and in the access routes of EMS-physicians. In order to investigate the success of establishment of palliative crisis cards associated with training and the rural and urban EMS structures, two urban and two rural EMS areas were compared using the Paramedic Palliative Care Test (PARPACT). **Methods:** The PARPACT includes test items on palliative knowledge (PK, maximum score: 15 points) and palliative self-efficacy expectations (PSE, maximum score: 18 points), as well as items on palliative attitudes in dealing with palliative care patients. We used a 4-point Likert-type scale. For data analysis, nonparametric tests (χ-test and Mann–Whitney *U* test) were used in addition to descriptive analysis (frequencies, means, medians, standard deviations, and ranges). **Results:** In total, 291 out of 750 ambulance or EMS personnel participated in the voluntary survey. Rural ambulance or EMS personnel answered the PK-questions correctly more often on average (mean: 11.19, SD: 1.85) than urban ambulance or EMS personnel (mean: 9.18, SD: 2.39; Mann–Whitney *U* test: *U*=5040.000, *P*=.001). In addition, ambulance or EMS personnel with the highest level of training (3-year-trained paramedics) performed better in PK (mean: 10.38, SD: 2.31) than less intensively training ambulance or EMS personnel (mean: 9.58, SD: 2.43; Mann-Whitney *U*-test: *U*=8446.500, *P*=.004). In terms of PSE, rural ambulance or EMS personnel also achieved higher mean PSE-scores (mean: 12.55, SD: 2.60) than urban ambulance or EMS personnel (mean: 9.77, SD: 3.41; Mann-Whitney *U*-test: *U*=5148.500, *P*=.001). **Conclusions:** Better training in the EMS is associated with improved PK compared to less qualified nonphysician EMS staff. The establishment of palliative crisis cards and the structures in the city alone do not lead to improved knowledge and PSE.

## Introduction

Palliative care is an important component of German Emergency medical service (EMS), as 3% of all EMS missions are related to palliative care patients.^1^ An EMS-physician is involved in the majority of these missions. Therefore it is very important to improve the knowledge of the nonphysician staff. Since many palliative care patients in Germany are also cared for at home by Specialized Outpatient Palliative Care (SOPC) Services, the importance of palliative knowledge (PK) and a palliative self-efficacy expectation has also increased in the EMS service.^2^ Despite established care structures for palliative care patients (eg SOPC) who are cared for at home, relatives still call the EMS in emergency situations or in case of symptom exacerbations.^3,4^ Previous studies have already shown that communication between relatives and EMS in particular needs to be improved (eg in terms of shared decision making).^5,6^ In order to assist EMS-teams in situations of acute emergencies, so-called palliative crisis and emergency cards have been introduced in several German cities, which are intended to provide EMS staff with a quick overview of the patient's will.^7,8^ Because EMS staff often feel pressured to make quick decisions so that ambulances can be freed up again being available for other cases.^5^ These palliative crisis cards were also established because advance directives are often formulated too vaguely.^7^ The proper establishment and use of the cards is usually linked to training for EMS staff. A study by Pease et al^9^ showed that palliative care training has positive effects.^9^ The training led to an increased self-confidence of the EMS staff in dealing with palliative care patients, particularly with regard to conversation and communication abilities. Carter et al^10^ also found that training can increase paramedics’ confidence in their own resources and improve the care of palliative care patients.

EMS in Germany is designed as a two-tiered system including physician-staffed EMS unit in life-threatening cases. EMS-physicians are usually not available as quickly in the countryside as in the city. In addition, EMS routes or transport to the nearest hospital are often longer in rural areas. However, an investigation of these differences has not yet been initiated. The aim of this study is to work out the differences between the urban and rural structured EMS with regard to PK and palliative self-efficacy expectations (PSE) in order to improve the quality of palliative care by the EMS. This study refers exclusively to nonmedical EMS personnel, who are usually first on the scene in rural areas to manage emergency situations, often without an EMS-physician. Using the Paramedic Palliative Care Test (PARPACT), a comparative analysis of two urban structured EMS areas with established palliative crisis or emergency card systems and two rural structured EMS areas without an established palliative crisis or emergency card system was carried out.^11^

## Methods

### Study Design

The study is a multicenter observational case-control study. The hypothesis was that the EMS staff in the urban structured EMS areas achieves a higher PK and a higher self-efficacy expectation than the EMS staff in the rural areas, possibly due to better training and the establishment of palliative crisis and emergency cards.

### Setting and Participants

The survey took place between March and December 2019. All ambulance or EMS personnel with at least 160 h of training and a final state examination were included in the survey. Ambulance or EMS personnel with shorter training periods and without a state examination as well as trainees and medical staff were excluded. In order to ensure good objectivity of the study, the questionnaire study with the EMS staff was conducted during regular training sessions in the EMS areas. EMS staff were interviewed in two large German cities, each with almost 600,000 inhabitants, and two German rural districts with 300,000 and 140,000 inhabitants, respectively. The urban EMS areas were characterized by a high hospital density with short travel distances to the next available emergency unit, a short arrival time of EMS-physicians at the scene, a well-established SOPC as well as a palliative crisis or emergency card systems established by means of training. The rural districts differ from the large cities not only in their lower hospital density, but also in the long distances they have to travel to the nearest hospitals, sometimes 30–40 min. The supply of EMS-physicians also means that in some cases they take much longer to arrive at the scene of the emergency than in large cities. SOPC, palliative care units and hospices are represented regionally in the rural districts (Table 1). Furthermore, a palliative crisis or emergency card system to support the EMS in rural areas has not yet been established. In addition to EMS-physicians, the German EMS mainly employs EMS staff with different training and competencies. There are different levels of training in Germany, with the “expert level” including 4600 h (3-year-trained paramedics), the “advanced level” including 2800 h, the “intermediate level” including 520 h and the “basic education level” including 160 h.

**Table 1.** Palliative Structures of the Individual EMS Areas in Direct Comparison.

	Duesseldorf	Essen	County Emsland	County Bentheim
Inhabitants^a^ (number in 2019)	619,294	583,109	325,657	136,511
University hospitals^b^ (number)	1	1	0	0
Hospitals (total)^b^ (number)	37	27	9	4
Palliative care units^c^ (number)	4	3	2	1
SOPC (number)^c^	2	1	2	1
Inpatient hospice^c^ (number)	2	3	1	0
Care services^c^ (number)	3	4	2	1

Abbreviations: EMS, emergency medical service; SOPC, specialized outpatient palliative care.

a source: Eurostat.

b source: https://www.kliniken.de.

c source: https://www.wegweiser-hospiz-palliativmedizin.de.

### The PARPACT-Questionnaire

The PARPACT was used in order to compare a rural and an urban structured EMS area.^11^ Items for PK, specific palliative self-efficacy expectations and for palliative attitudes in dealing with palliative care patients were included. Palliative attitude was defined as the perception of EMS staff regarding the importance, concerns, and opinions on the feasibility of palliative care in the respective EMS areas. The term palliative unit was defined for inpatient and/or (SOPC) or collaboration in a hospice movement. For each item, a 4-point Likert-type scale with the categories (“strongly agree,” “agree,” “disagree,” and “strongly disagree”) was used. Overall, a maximum PK-Score of 15 points could be achieved. A maximum PSE-Score of 18 points could be achieved in this section. The maximum achievable palliative attitude score was set at 4 points per item.

### Statistical Analysis

The data were analyzed using SPSS version 25 (IBM, Armonk, NY, USA). Frequencies, percentages, means, medians, standard deviations, and ranges were calculated for the descriptive analysis. Before selecting the appropriate tests for the analysis, the data were checked for normal distributions using the Kolmogorov–Smirnov test. Due to significant deviations from a normal distribution, we used the Mann–Whitney *U* test to explore differences in the PARPACT-scores. χ^2^-tests were further used to assess whether there are differences in demographics. A significance level of *P*<.05 was set for this. For the correlation analyses, the Spearman correlation coefficient was used.

### Ethical Considerations

The EMS staff were informed about the objectives, the voluntary nature of participation and the data protection regulations of the survey. After having given informed consent, participants were given the respective, nonpersonalized questionnaires. The local ethics committee approved the study.

## Results

Between March 2019 and December 2019, a total of 291 of 750 ambulance or EMS personnel from Duesseldorf, Essen, County Emsland, and County Bentheim participated in the voluntary survey. This represents a recruitment rate of 39%. 181 EMS staff worked in an EMS responsible for the city and 110 of the respondents were employed in an EMS responsible for the rural area.

The Kolmogorov–Smirnov test did not show a normal distribution for any of the items examined (P< .001).

### Participant Characteristics

The demographic data are shown in Table 2. On average, urban ambulance or EMS personnel were older than the rural ambulance or EMS personnel (37 vs 32 years). In addition, urban ambulance or EMS personnel were on average more experienced (13 vs 9 years) and more often male overall (97% vs 67%). In both urban and rural areas, almost half (44% vs 48%) of the participating ambulance or EMS personnel are 3-year-trained paramedics. Both 3% of the urban and rural ambulance or EMS personnel reported having worked in a palliative care unit.

**Table 2.** Demographics of Urban and Rural Ambulance or Emergency Medical Service (EMS) Personnel.

	Total, n=291	EMS* Urban, n=181	EMS* Rural, n=110	*P* value
Gender, [n (%)]				.001^a^
Female	37 (13%)	4 (2%)	33 (30%)	
Male	294 (86%)	175 (97%)	74 (67%)	
Divers	1 -	1 -	0 -	
No answer	4 (1%)	1 -	3 (3%)	
Age, [years]				.001^b^
Mean (SD)	35 (9.4)	37 (8.5)	32 (10)	
Range	18-60	21-60	18-60	
Work experience, [years]				.001^b^
Mean (SD)	11 (8.2)	13 (8.4)	9 (7.4)	
Range	1-35	1-35	1-32	
Level of training [n (%)]				
Expert level^c^	132 (45%)	79 (44%)	53 (48%)	.004^a^
Advanced level^d^	68 (23%)	50 (28%)	18 (16%)	
Intermediate level^e^	85 (29%)	52 (28%)	33 (30%)	
Basic education level^f^	2 (1%)	-	2 (2%)	
No answer	4 (2%)	-	4 (4%)	
Number of interventions with palliative care patients [per year]				
<5	108 (37%)	80 (44%)	28 (26%)	.002^a^
5-10	107 (37%)	62 (34%)	45 (41%)	
10-20	50 (17%)	23(13%)	27 (25%)	
21-50	17 (6%)	8 (4%)	9 (8%)	
>50	6 (2%)	5 (3%)	1 -	
No answer	3 (1%)	3 (2%)	-	
Have already worked in a palliative care unit [n (%)]				
Yes	8 (3%)	5 (3%)	3 (3%)	1.000^a^
No	283 (97%)	176 (97%)	107 (97%)	

*ambulance or EMS personnel.

a Chi-square test (exact values are calculated).

b Mann-Whitney *U* test.

c Expert level—4600 h of training and state examination (3-year-trained paramedics).

d Advanced level—2800 h of training and state examination.

e Intermediate level—520 h of training and state examination.

f Basic education level—160 h of training and state examination.

### Palliative Knowledge

**Figure 1.**
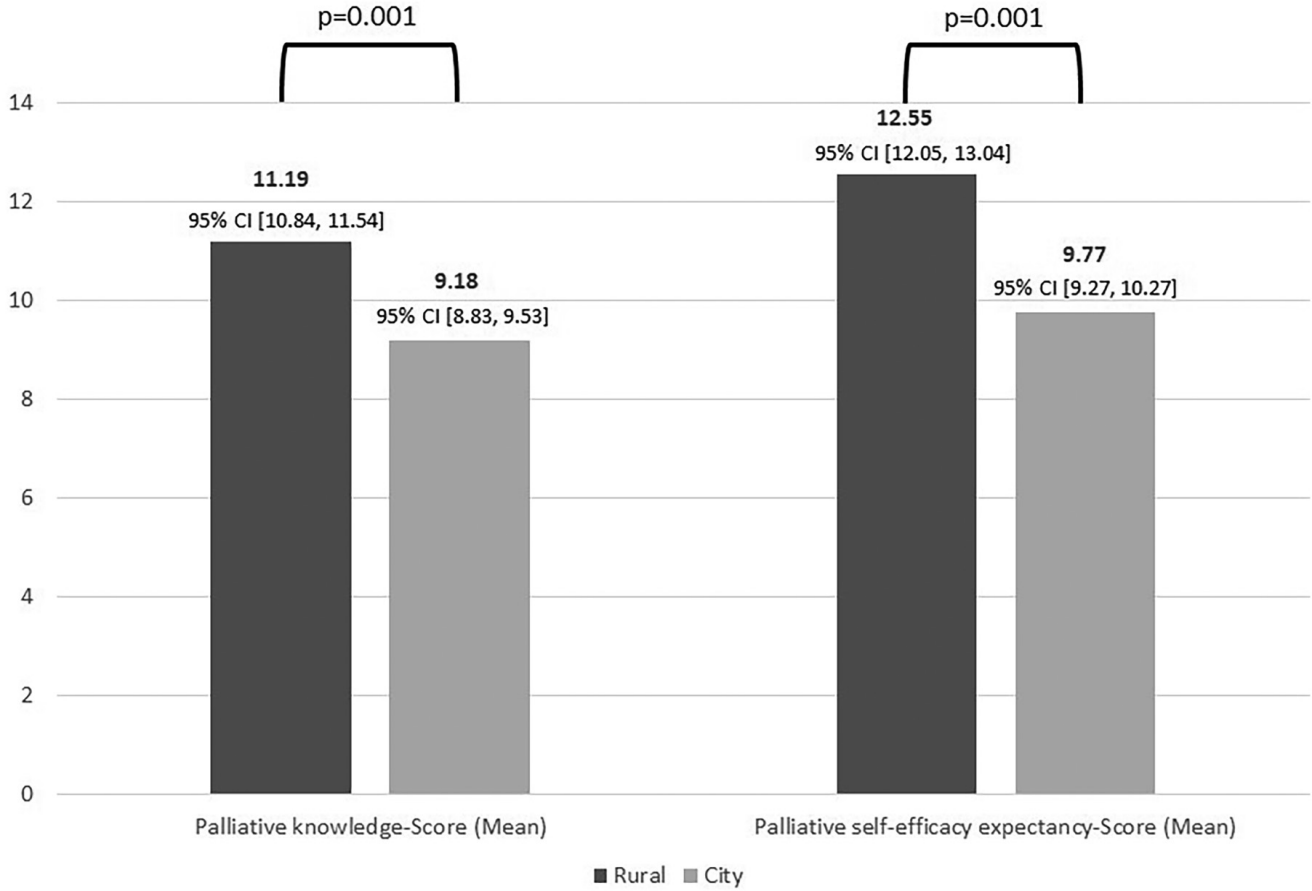
Palliative knowledge score and palliative self-efficacy expectancy score of urban and rural ambulance or emergency medical service (EMS) personnel. CI = Confidence Interval.

Rural ambulance or EMS personnel answered the questions on PK correctly more often (mean: 11.19, SD: 1.85) than urban ambulance or EMS personnel (mean: 9.18, SD: 2.39; Mann-Whitney *U* test: *U*=5040.000, *P*=.001, *r* = 0.42) (Figure 1**)**. The effect size for this difference is *r* = 0.42, which could be—in accordance with Cohen (1988)—interpreted as a moderate to large effect size.^12^ As can be seen in Table 3, the ambulance or EMS personnel performed better in detail when answering questions on pain and dyspnea treatment and on legal topics. With regard to the topics “euthanasia” and “communication and end of life care,” there were no significant differences. Further analysis also indicated that ambulance or EMS personnel who had already completed the 3-year training (expert level) answered questions on PK correctly more often on average (mean: 10.38, SD: 2.31) than less intensively trained ambulance or EMS personnel (mean: 9.58, SD: 2.43; Mann-Whitney *U*-test: *U*=8446.500, *P*=.004). Similarly, ambulance or EMS personnel with reportedly between 21–50 palliative care calls per year answered more questions correctly (mean: 10.94, SD: 2.56) than ambulance or EMS personnel with less than five calls (mean: 9.58, SD: 2.64; Mann-Whitney *U*-test: *U*=634.000, *P*=.039). There was no significant difference in PK between ambulance or EMS personnel who had already worked in a palliative care unit and those without previous palliative care experience (Mann-Whitney *U*-test: *U*= 1115.000, *P*=.942).

**Table 3.** Differences in Responses to Items of Palliative Care Topics Between Urban and Rural Ambulance or Emergency Medical Service (EMS) Personnel.

Topics	EMS^a^ Urban, mean (SD)	EMS^a^ Rural, mean (SD)	*P* value
Pain and dyspnea treatment	1.35 (0.93)	1.89 (0.90)	.001^b^
Legal topic	1.45 (0.55)	1.71 (0.48)	.001^b^
Communication and end-of-life care	1.96 (0.21)	1.97 (0.16)	.453^b^
Palliative care structures	2.58 (0.70)	2.82 (0.41)	.005^b^
Active end-of-life care	0.62 (0.49)	0.64 (0.48)	.816^b^

a ambulance or EMS personnel.

b Mann–Whitney *U* test.

### Palliative Self-Efficacy Expectation

On average, rural ambulance or EMS personnel achieved a higher PSE-Score (mean: 12.55, SD: 2.60) than urban ambulance or EMS personnel (mean: 9.77, SD: 3.41; Mann-Whitney *U*-test: *U*=5148.500, *P*=.001) **(**Figure 1**)**. In the evaluation, a medium effect (*r* = 0.41) could be demonstrated for this significant difference. A correlation between the higher PK and the higher palliative self-efficacy expectation of the rural ambulance or EMS personnel could not be determined (Spearman correlation *r* = 0.056, *P*=.564). Overall, there was no significantly higher self-efficacy expectation of ambulance or EMS personnel with completed the 3-year training (“expert level,” mean: 10.74, SD: 3.25), compared to ambulance or EMS personnel with lower professional qualifications (mean: 10.89, SD: 3.53; Mann-Whitney *U*-test: *U*=9947.500, *P*=.442). The number of assignments with palliative care patients also had an influence on the self-efficacy expectations of ambulance or EMS personnel. Ambulance or EMS personnel with more than 21 palliative care calls per year felt significantly more confident (mean: 12.65, SD: 2.89) than ambulance or EMS personnel with less than five palliative care calls per year (mean: 9.97, SD: 3.52; Mann-Whitney *U*-test: *U*=508.000, *P*=.003). No significant differences in self-efficacy expectations were found between ambulance or EMS personnel who had already worked in a palliative care unit and those without previous experience in palliative care (Mann-Whitney *U*-test: *U*=1050.000, *P*=.726).

### Palliative Attitude

Both rural and urban ambulance or EMS personnel agreed that it made sense for palliative care patients to create an advance directive (Mann-Whitney *U*-test: *U*=9325.000, *P*=.312). From the point of view of both groups, palliative care patients should also clearly state their position on hospital treatment in their advance directives (Mann-Whiney *U*-test: *U*=25326.000, *P*=.267). There was also consensus that having a palliative crisis or emergency card system was important for palliative care patients (Mann-Whitney *U*-test: *U*=9553.000, *P*=.648). However, the two groups differed in their opinion on the consideration of the patient's will recorded in advance directives. In contrast to urban ambulance or EMS personnel (mean: 2.72, SD: 0.99), rural ambulance or EMS personnel (mean: 3.19, SD: 0.81) stated that their actions at the scene were more often influenced by advance directives (Mann-Whitney *U*-test: *U*=7191.500, *P*=.001).

## Discussion

Several publications have already pointed out the importance of good education and training in palliative care for EMS staff.^13–15^ Since many palliative care patients are now cared for at home, the importance of good training in palliative medicine is of great significance order to be sufficiently competent in palliative care missions. To the best of our knowledge, no study has investigated the PK and self-efficacy expectations of ambulance or EMS personnel using a validated measurement instrument.

Despite an established palliative crisis or emergency card and associated training, in our study, the rural EMS staff showed higher PK and self-efficacy expectations. In particular, rural ambulance or EMS personnel also performed better in answering questions on pain or dyspnea management. Not only since the introduction of the highest level of training in the German EMS in 2014 have EMS staff been given more competencies. For example, within the framework of standardized operating procedures (SOPs) certain medications may be administered by nonmedical staff in the German EMS after strict indication has been established.^16,17^ This fact could offer an opportunity to improve the conflict faced by EMS which are more likely to take patients to hospital after administering medication due to a lack of practice guidelines in some cases.^5^ It is already well known that SOPs optimize the care of emergency patients in the EMS and they are therefore used in the various EMS systems worldwide.^18,19^ SOPs can be of great advantage in the treatment of palliative care patients both in the hospital and in the EMS.^20,21^ The increased PK in the management of pain and dyspnea may also be explained by the increased independent handling of medications or their more frequent use, as a result of delayed arrival and less frequent involvement of an EMS-physician in rural areas. The finding that dyspnea and pain are among the most frequent reasons for calling an EMS emphasizes its centrality to the day-to-day work in EMS.^4,5^ Furthermore, Surakka et al^4^ found that the EMS in Finland is called out more often for palliative care patients in rural areas than in urban areas. More frequent use with palliative care patients could also be another explanation for the finding that rural ambulance or EMS personnel scored better in PK than urban ambulance or EMS personnel. However, there are relevant differences in the worldwide EMS systems. Thus, a two-tiered physician-staffed EMS system, which is used in Germany, cannot be compared across the board with the EMS system based on paramedics, which is used in many Anglo-American countries. In paramedic-based EMS, medications are usually administered less frequently in emergency situations. In addition, it has been pointed out in the past that paramedics feel more insecure about complying with advance directives and Do not attempt resuscitation orders for legal reasons.^22,23^

Almost half of all participating EMS staff are 3-year-trained paramedics and had the highest possible level of training in the German EMS. In our study, the 3-year-trained paramedics answered more questions in PK correctly than the less qualified staff. This result reflects the gain in competence through intensified training and is also in line with the findings of previous studies in Germany and Australia.^7,15^

Another difference between urban and rural EMS was found in the knowledge of palliative care structures. Here, too, the rural EMS staff performed better. Often there is only insufficient knowledge of the care structures. However, knowledge of these structures is important in order to be able to implement the requirements for professional care coordination in palliative care.^5^

The ambulance or EMS personnel with allegedly between 21 and 50 assignments per year with palliative care patients, answered significantly more questions in PK correctly an achieved higher PSE-Scores than the ambulance or EMS personnel with less than 5 assignments. Although the data on the number of interventions with palliative care patients were subjective estimates of the EMS, it can be assumed that increased contact with palliative care patient also leads to higher PK and increased self-efficacy expectations. Kirk et al^14^ could show that in the English EMS self-confidence in missions with palliative care patients increases with growing experience.

However, there was no difference in the responses to items on the psycho-social care of palliative care patients. Meeting the psycho-social needs of patients in palliative care is another requirement placed on EMS staff.^15,24,25^ Especially in the case of time-relevant issues, the EMS staff often has to make decisions in the best interest of the patient before the EMS-physician arrives. It is already known that the rural EMS is called more often for palliative care patients, but the influence of this fact on the self-efficacy expectations of rural EMS staff has not yet been investigated.^4^ Previous studies have also shown that experienced paramedics generally feel more confident in dealing with palliative care patients.^1,2,26^ In contrast, the rural staff in our study were on average less experienced, but still felt more confident in caring for palliative care patients than the urban staff.^1,2,26^ The increasing introduction of telemedicine means that EMS personnel have an additional opportunity to consult with physician staff. This has already led to a proven reduction in transports to urban emergency departments.^27^ Furthermore, it can be assumed that every opportunity for consultation leads to an increased feeling of security among emergency services staff when they leave patients at the scene. However, our study showed that rural EMS personnel feel more confident when dealing with palliative care patients, which may be due to the fact that they have to treat patients alone and make decisions before the EMS-physician arrives. The need for rural EMS staff to make relevant decisions at an early stage is also evident in the attitudes of rural EMS staff toward considering the patient's wishes. All EMS staff agree that it is useful to have palliative crises cards or an advance directive. Our research additionally showed that, in comparison to previous research in Germany and England, rural EMS staff in particular believe that the content of these directives influences their actions at the scene of an emergency.^7,28^ A circumstance that also suggests that the EMS staff in the city rely primarily on the assessment of the EMS-physicians, who often arrive at the scene at the same time as the ambulance or are quickly available. These decisions once again underline the importance of palliative care training. Paramedics are also more likely to be familiar with life-sustaining measures due to a lack of palliative care training.^4^ However, optimized protocols for EMS could improve home-based care for palliative care patients.^4^ As Verhoef et al^29^ and Gage et al^6^ have noted in the Netherlands and South Africa, early recognition of palliative situations may prevent hospitalization, which is not in accordance with the patient's wishes.

### Limitations

Since the survey of ambulance or EMS personnel in our study took place on a voluntary basis within the framework of general paramedics training, it can be assumed that predominantly motivated ambulance or EMS personnel with an interest in palliative care topics took part in the survey. This fact is also reflected in the recruitment rate of 39%. Furthermore, the proportion of women in the EMS is significantly higher in rural areas than in urban areas. Therefore, this study is not comparable to the population of all ambulance or EMS personnel working and further research should follow. It should also be mentioned that EMS systems internationally cannot be compared across the board due to their different structures.

## Conclusions

This study is the first investigation of PK and palliative self-efficacy expectations among German ambulance or EMS personnel. It was able to show 3-year-trained paramedics (expert level) lead to improved PK compared to less qualified ambulance or EMS personnel. According to our research, the establishment of palliative crisis and emergency card systems combined with training and the structures in the city on their own do not lead to improved knowledge and self-efficacy expectation. Rather, it seems that above all the greater autonomy of rural ambulance or EMS personnel influences PK and palliative self-efficacy expectation.
